# Fungal Quorum-Sensing Molecules: A Review of Their Antifungal Effect against *Candida* Biofilms

**DOI:** 10.3390/jof6030099

**Published:** 2020-07-02

**Authors:** Renátó Kovács, László Majoros

**Affiliations:** 1 Department of Medical Microbiology, Faculty of Medicine, University of Debrecen, 4032 Debrecen, Hungary; major@med.unideb.hu; 2 Faculty of Pharmacy, University of Debrecen, 4032 Debrecen, Hungary

**Keywords:** *Candida*, farnesol, tyrosol, biofilm, therapy, combination

## Abstract

The number of effective therapeutic strategies against biofilms is limited; development of novel therapies is urgently needed to treat a variety of biofilm-associated infections. Quorum sensing is a special form of microbial cell-to-cell communication that is responsible for the release of numerous extracellular molecules, whose concentration is proportional with cell density. *Candida*-secreted quorum-sensing molecules (i.e., farnesol and tyrosol) have a pivotal role in morphogenesis, biofilm formation, and virulence. Farnesol can mediate the hyphae-to-yeast transition, while tyrosol has the opposite effect of inducing transition from the yeast to hyphal form. A number of questions regarding *Candida* quorum sensing remain to be addressed; nevertheless, the literature shows that farnesol and tyrosol possess remarkable antifungal and anti-biofilm effect at supraphysiological concentration. Furthermore, previous in vitro and in vivo data suggest that they may have a potent adjuvant effect in combination with certain traditional antifungal agents. This review discusses the most promising farnesol- and tyrosol-based in vitro and in vivo results, which may be a foundation for future development of novel therapeutic strategies to combat *Candida* biofilms.

## 1. Introduction

It has been estimated that there are 2.2 to 3.8 million fungal species worldwide; however, approximately 300 species have been described to cause human disease [1]. *Candida* species are among the most common human fungal pathogens. The annual incidence rate of *Candida*-associated bloodstream infections ranged from 9.5 to 14.4 per 100,000 in the United States of America [2]. This value ranged from 1.4 to 5.7 per 100,000 in Europe, depending on the country [3]. In the last two decades, the prevalence of resistant fungal infections has been steadily increasing due to the widespread use of antifungals in agriculture and veterinary and human medicine [4,5]. Global warming and anthropogenic effects have resulted in the emergence of previously little-known, potentially multi-resistant fungal pathogens in clinical practice, such as *Candida auris*, azole-resistant *Aspergillus* spp., or *Lomentospora prolificans*. These emerging pathogens have caused further challenges for therapy [6,7].

Several fungal species can switch their morphology from yeast to hyphal or pseudohyphal forms, which is coupled with biofilm formation and plays a pivotal role both in fungal virulence and in resistance to antifungals [8,9,10]. The increased number of biofilm-associated infections is exacerbated by a paucity of antifungal agents or therapeutic strategies in development that have unique mechanisms of action or possess alternative approaches, respectively [11]. Currently, the most promising antifungal agents are already in Phase 3 including ibrexafungerp [12], rezafungin [13], super bioavailable itraconazole [14], and VT-1161 [15]. Recently investigated alternative therapeutic approaches involve high-dose therapy with available antifungal agents [16,17,18], antifungal lock therapy [19], and combination-based therapies [20,21]. Based on in vitro and in vivo data, echinocandins and amphotericin B solutions are the most promising combination-based and/or antifungal lock strategies [19]. Further innovative therapeutic approaches may be the natural products-based treatments [22,23]. One of the more well-studied compounds is carbohydrate-derived fulvic acid as a heat stable colloidal material, which has an inhibitory effect on *Candida* and bacterial biofilm formation [24]. Moreover, a further alternative approach is the treatments disrupting quorum sensing. The usage of quorum sensing molecules at supraphysiological concentration may adversely influence the cell-to-cell communication in biofilms [25,26,27]. In addition, the quorum-sensing system can be inactivated, which is generally known as quorum quenching. Quorum quenching can be triggered by inhibiting the production of quorum sensing molecules, their detection by receptors or their degradation [28].

In this review, a detailed overview is provided of the recent status of quorum-sensing molecule-based therapeutic approaches and their potential future perspectives against *Candida* biofilms.

## 2. The Medical Importance of *Candida* Biofilms

Despite their importance, *Candida* biofilms remain a relatively underappreciated and understudied area. Therefore, effective therapeutic strategies against these sessile communities remain scarce. Biofilms are usually found in medical devices such as joint prostheses, pacemakers, urinary and central venous catheters, dentures, and mechanical heart valves, hindering the eradication of *Candida* infections [10]. In addition, several chronic *Candida*-related diseases are also associated with biofilm development [29]. Biofilm formation on the vaginal mucosa has been observed in in vivo models of vulvovaginal candidiasis [30]. Oral- and oesophageal mucosae-associated biofilms are a very important contributor to oral diseases caused by *Candida* species; gastrointestinal and urogenital tracts are also common sites of *Candida*-associated opportunistic infections [31]. *Candida* is one of the most commonly identified fungal genera in wounds whose environment can also promote the formation of biofilms [32]. A series of recent studies has indicated that strains defective in hyphal formation display significantly milder symptoms, highlighting the role of biofilm formation in pathogenesis of these chronic or recurrent infections [30,33].

These sessile communities exhibit five- to eightfold higher resistance to all licenced antifungal drugs when compared to their planktonic counterparts [10]. This high rate of resistance can be explained by the increased metabolic activity of cells in the early development phase of biofilm formation [10]. On the other hand, dormant, non-proliferating persister cells have also been observed, especially in mature biofilms, that have demonstrated high tolerance to antifungals [34]. Furthermore, the various *Candida* species can produce dense extracellular polymeric substances which serve as a solid barrier to prevent the diffusion of antifungal drugs and account for almost 90% of the biofilm dry mass [10]. As has been previously reported in the literature, sessile *Candida* communities exhibit an altered gene expression profile, including the upregulation of *CDR* and *MDR* genes which encode azole resistance transporter proteins, and pose further challenges for treatment [35].

To date, there is no definitive therapy against *Candida* biofilms; nevertheless, there are several promising in vitro, in vivo and clinical results. The increasing number of resistant *Candida* species and isolates highlight the need for new molecules with new targets. Alternative therapeutic approaches against multidrug-resistant fungal biofilms may be the result of a combination of traditional antifungal agents with quorum-sensing molecules [36].

## 3. Fungal Quorum Sensing

A major mechanism of microbial communication is a population density-dependent stimulus-response system called quorum sensing. This process occurs by the continuous release and monitoring of low molecular weight hormone-like secreted molecules (quorum-sensing molecules), which are not elementary in the central metabolism but have a variety of biological activities. The concentration of these quorum-sensing molecules is proportional with the size of population; after reaching a critical threshold, a response is triggered leading to the coordinated expression or repression of quorum sensing-related target genes [37].

In the fungal kingdom, quorum sensing was a relatively unknown phenomenon until Hornby et al. (2001) described the effect of the isoprenoid farnesol on *Candida albicans* morphogenesis; this opened a new branch of science focusing on fungal quorum sensing [38]. At the same time, quorum sensing has been already reported in *Aspergillus* spp. [39] and *Penicillium* spp. [40]. To date, four main quorum-sensing molecules were described including farnesol, tyrosol, phenylethanol, and tryptophol, which have a remarkable effect on the regulation of morphogenesis (yeast to hyphae transition and vice versa), initiation of fungal apoptosis, and virulence [41]. 

Recently, several authors reported that certain quorum-sensing molecules may generate oxidative stress, especially at supraphysiological concentrations, which may have an antifungal effect [42,43,44,45]. The majority of data concerning fungal quorum sensing molecule-related therapeutic potential derived from *C. albicans* experiments, and these results cannot be always directly extrapolated to non-*albicans* species. Recently, the number of studies dealing with the effect of quorum-sensing molecules on non-*albicans* species has steadily increased, supporting the comprehensive understanding of the in vitro and in vivo antifungal effects exerted by these molecules. 

## 4. Farnesol

### 4.1. Physiological Effect of Farnesol in Candida Species

Farnesol (3,7,11-trimethyl-2,6,10-dodecatriene-1-ol) was the first described *Candida*-derived quorum sensing molecule; it is released in *C. albicans* as a side product of the sterol synthetic pathway by dephosphorylation of farnesol pyrophosphate [38,46]. It is an acyclic sesquiterpene heat-stable molecule, which is produced primarily under aerobic conditions and it is unaffected by extreme pH and the type of carbon or nitrogen source [38,47]. Generally, the farnesol concentration is proportional to the colony-forming unit number [38]. Under physiological conditions, *C. albicans* isolates secrete a farnesol concentration with a mean of 35.6 μM (range: 13.7 to 58.5 μM) [48]. This concentration was 35 times higher than that secreted by non-*albicans* species, with the exception of *Candida dubliniensis*, which has demonstrated a concentration of 8.3 μM (range: 6.0 to 17.5 μM). All other non-albicans species excreted significantly lower farnesol concentrations, ranging from 0.4 to 1 μM [48]. These differences in excretion may be explained by the species-specific characteristics in sterol synthesis [49].

Based on a cDNA microarray analysis, a total of 274 genes were identified as responsive in *C. albicans*, with 104 genes up-regulated and 170 genes down-regulated [50]. Farnesol has an ability to influence *Candida* morphology, biofilm formation, drug efflux pump expression, apoptosis regulation, phagocytic response, surface hydrophobicity, iron metabolism, and heat-shock-related pathways [50,51,52,53,54]. One of the most prominent farnesol-associated effects is the induction of hypha-to-yeast transition and the inhibition of biofilm formation in various *Candida* species. It should be emphasized that 150-fold more farnesol is needed to block germ-tube formation in the presence of 10% serum, showing that it can bind to serum proteins at a high rate [55,56].

In view of this diverse role, it is not surprising that this compound influences several central signalling pathways in different *Candida* species. One of the best-studied farnesol-related pathways is the Ras1-cAMP-PKA cascade, where farnesol binds to the cyclase domain of the adenylyl cyclase *Cyr1*, influencing the level of intracellular cAMP [57]. Moreover, farnesol induces the cleavage of the small GTPase Ras1, resulting in a soluble Ras1; soluble Ras1 is a weak activator of *Cyr1* and supports the formation of yeast cells [58]. Furthermore, farnesol can directly inhibit the cAMP signalling pathway, supporting the hypha-to-yeast transition [59]. It is noteworthy that farnesol exposure stabilizes the *Nrg1* protein, which is the negative regulator of filamentation [60]. While farnesol was described first in *C. albicans*, it can inhibit filamentation and growth in other fungal species [27,61], including *Saccharomyces cerevisiae* [62], *Aspergillus niger* [63], *Aspergillus flavus* [64], *Aspergillus nidulans* [65], *Penicillium expansum* [66], *Fusarium graminearum* [67], and *Paracoccidioides brasiliensis* [68].

Regarding reactive oxygen species production, the supraphysiological farnesol concentrations (200–300 μM) are stressful for most fungi, while the physiological concentrations (30–40 μM) protect them from stress [57]. In addition to the farnesol-related effect on growth in the case of different microbes, the molecule also has a relevant immunomodulator effect [57,69]. Farnesol can stimulate both macrophage chemokine synthesis or macrophage recruitment, and trigger activation of neutrophil granulocytes and monocytes. Farnesol exposure also influences the differentiation of monocytes into dendritic cells [57,69].

Farnesol has been reported to induce cell growth inhibition and/or apoptosis in tumor cells where the observed IC_50_ values varied widely for different tumor types and different cell lines [70]. Farnesol caused 100% cell death at >120 μM in A549 and H460 lung cancer cells [71]. Scheper et al. (2008) observed an IC_50_ value of 30 to 60 μM for farnesol on the primary human tongue squamous cell carcinoma cell lines (OSCC9, OSCC 25) [70]. Nagy et al. (2020) evaluated 10 μM, 50 μM, 150 μM, and 300 μM farnesol concentrations in terms of toxicity to the Caco-2 cell line, where no toxicity was observed with any concentration tested [45].

### 4.2. Antimicrobial Activity of Farnesol

At physiological concentrations, farnesol has no significant effect on *Candida* cells that have already begun hyphae development or biofilm formation [25,38]. However, prior results suggest that farnesol can cause biofilm degradation at supraphysiological concentrations, suggesting the potential use of this compound in biofilm-associated infections [36]. In addition, several authors have published studies demonstrating contribution of farnesol to reduced azole resistance of *Candida* cells, including in biofilms [72]. This phenomenon can be explained by the modulation of *Cdr1* efflux pumps, reactive oxygen species production, or changes in glutathione homeostasis [38,61,72]. Furthermore, farnesol has an effect on genes connected to ergosterol synthesis [46]. Dižová et al. (2018) observed that the presence of 200 μM farnesol down-regulated the *ERG20*, *ERG11* and *ERG9* genes. However, this farnesol concentration supplemented with 0.5 mg/L fluconazole restored the original expression level of *ERG20* and *ERG11*. Interestingly, the physiological farnesol concentration (~30 μM) only slightly influences the expression of these genes in 48 h-old biofilms [73]. Chen et al. (2018) reported that *CYR1* and *PDE2* regulate resistance mechanisms against various antifungals in *C. albicans* biofilms. However, farnesol can diminish the resistance of *C. albicans* biofilms by regulating the expression of the gene *CYR1* and *PDE2* [74]. Yu et al. (2012) observed that the sterol biosynthetic pathway may contribute to the inhibitory effects of farnesol, as the transcription levels of the *ERG11*, *ERG25*, *ERG6*, *ERG3*, and *ERG1* genes decreased following farnesol exposure [75]. Jabra-Rizk et al. (2006) showed that farnesol concentrations of 30–50 mM decrease the fluconazole MICs for *C. albicans* and *C. dubliniensis* from resistant values to a susceptible dose-dependent range, while concentrations of 100–300 mM resulted in fluconazole susceptibility [76].

One of the first major breakthroughs in combination-based experiments with farnesol and antifungals was published by Katragkou et al. (2015), who found a significant synergy against *C. albicans* 48 h-old biofilms between fluconazole, amphotericin B, and micafungin in the presence of farnesol [26]. The highest synergistic effect was observed in the case of micafungin combined with farnesol using fractional inhibitory concentration index determination and Bliss independence analysis. Based on the Bliss model, the observed effects were 39–52% higher compared to the expected efficacy if the drugs had been acting independently [26]. It should be noted that synergism was observed only in the case of farnesol/micafungin and farnesol/fluconazole based on calculated fractional inhibitory concentration indices, suggesting the usage of multiple analytical approaches for investigation of drug-drug interaction [26].

Regarding non-albicans species, Kovács et al. (2016) showed that farnesol consistently enhanced the activity of caspofungin and micafungin, as concordantly shown in two independent experimental settings (chequerboard dilution and time–kill experiments) [27]. Fernández-Rivero et al. (2017) reported that a supraphysiological farnesol concentration (300 μM) improved the activity of amphotericin B against *Candida tropicalis* biofilms but did not affect anidulafungin [77]. Two recent studies by Nagy et al. concluded that farnesol significantly enhanced the activity of echinocandins and triazoles against one-day-old *C. auris* biofilms in vitro, suggesting an alternative approach to overcome the previously well-documented azole and echinocandin resistance of *C. auris* biofilms [45,78].

Animal experiments with farnesol raised several questions in terms of in vivo applicability of this compound. In one of the first in vivo studies, Navarathna et al. (2007) concluded that the physiological farnesol production may play a pivotal role as a virulence factor in fungal pathogenesis; furthermore, exogenous oral and intraperitoneal farnesol administration (20 mM) enhances the mortality of mice in their systemic mouse model [79]. Contrary to these results, Hisajima et al. (2008) observed a protective effect against *C. albicans* in their oral candidiasis mouse model [80]. It should be noted that there was a 1000-fold difference between the administered farnesol dosages (9 μM/mouse) in the experiments of Hisajima et al. (2008) [80] compared to experiments performed by Navarathna et al. (2007) (20 mM/mouse) [79]. In addition, they reported a potential gastrointestinal tract-related farnesol effect including moderate bodyweight reduction and reduced *Candida* faeces burden [80]. A cocktail of *Candida*-derived regulatory alcohols combined with nanomolar amounts of farnesol was reported to have a similar protective effect by Martins et al. (2012) in their murine model of disseminated candidiasis [81]. Bozó et al. (2016) did not find a farnesol-related protective effect against vaginal *C. albicans* infection [82], in contrast to the findings of Hisajima et al. (2008) [80]. However, both administered farnesol regimens enhanced the activity of 5 mg/kg daily fluconazole treatment against fluconazole-resistant *C. albicans* strain [82]. Similar fluconazole resistance reversion was observed in the case of planktonic cells by Jabra-Rizk et al. (2006) [76] and Cordeiro et al. (2013) [83]. Fernandes Costa et al. (2019) used nanoparticles containing farnesol alone or in combination with miconazole; nanoparticles containing farnesol inhibited yeast-to-hyphae transition at concentrations greater than or equal to 240 μM [84]. In addition, chitosan nanoparticles containing miconazole (33 mg/L) and farnesol (2.1 mM) inhibited fungal proliferation and decreased the pathogenicity of mouse vulvovaginitis infection [84]. Nagy et al. (2020) demonstrated that a daily treatment with 75 μM farnesol decreased the *C. auris* kidney burden in their immunocompromised systemic mouse model, especially when inocula was pre-exposed to farnesol [45].

The farnesol-exerted antifungal activity can be explained by the higher level of reactive oxygen species, especially in the case of non-*albicans* species [43,45]. Furthermore, farnesol has an amphiphilic property which allows for its integration into cell membranes, influencing membrane fluidity and integrity. In the case of *Candida parapsilosis* and *C. dubliniensis*, farnesol affected the cellular polarization and membrane permeability [61,76,85]. These observations can help further elucidate the antifungal effect.

Farnesol has a remarkable antibacterial effect alone or in combination with traditional antibacterial agents as demonstrated by in vitro investigations. Jabra-Rizk et al. (2006) observed that farnesol treatment (100 μM) increases the activity of gentamicin against *Staphylococcus aureus* biofilms [86]. Gomes et al. (2009) showed that farnesol exposure (300 μM) produced a relatively long post-antimicrobial effect (>8 h) against *Staphylococcus epidermidis* [87], while Pammi et al. (2011) observed that farnesol exposure at a concentration of 500 μM significantly inhibited the *S. epidermidis* biofilm formation in vitro [88]. A clear synergistic interaction was observed between farnesol and nafcillin or vancomycin against *S. epidermidis* sessile cells [88]. Additionally, it potentiates the activity of beta-lactam antibiotics against antibiotic-resistant bacterium species [89]. Castelo-Branco et al. (2012) showed a potent antimicrobial effect exerted by exogenous farnesol exposure against mature *Burkholderia pseudomallei* biofilms [90]. Additionally, it increased the activity of amoxicillin, ceftazidime, doxycycline, and sulfamethoxazole-trimethoprim, which are routinely administered for the treatment of melioidoses [91]. Farnesol also had a synergizing effect against ciprofloxacin-resistant *Pseudomonas aeruginosa* biofilms when used in combination with ciprofloxacin [92]. In vivo data also supports the antibacterial efficacy of farnesol. It has been observed that 6.7 mM farnesol treatment significantly decreased the *S. epidermidis* associated catheter infection and systemic dissemination [88].

Based on several studies, farnesol has a remarkable effect in *Candida*-bacterium mixed biofilms. *C. albicans*-derived farnesol has also been shown to have an effect on the response of *S. aureus* to antibiotics in mixed species biofilms. Farnesol exposure results in a significant decrease in staphyloxantin, which is an important virulence factor of this bacterium [42]. Černáková et al. (2018) showed that 200 μM farnesol has an inhibitory effect on *C. albicans* growth in mixed-species biofilms with *Streptococcus mutans* [93]. Cugini et al. (2010) examined the *C. albicans*-*P. aeruginosa* mixed species biofilms, where the *C. albicans*-derived farnesol enhanced *P. aeruginosa* quinolone signal production in a LasR-defective strain [94].

## 5. Tyrosol

### 5.1. Physiological Effect of Tyrosol in Candida Species

Tyrosol (2-(4-hydoxyphenyl)-ethanol) is a tyrosine-derived molecule which is synthetized via either tyramine or 4-hydroxyphenylacetaldehyde [95,96]. In the case of *C. albicans*, it is released into the growth medium continuously during the exponential growth phase and is capable of decreasing the duration of the lag phase before cells begin germination. The accumulation of tyrosol in the culture medium is proportional to the rise of fungal cell number. While the molecule stimulates filamentation, it exclusively promotes germ tube formation in conditions that normally induce these physiological processes [95,96]. Tyrosol exposure influences cell cycle regulation, DNA replication, and chromosome segregation in *C. albicans* [95]. Additionally, it was shown to have an inhibitory effect on neutrophil granulocytes by interfering with the oxidative stress response of these phagocytes [97,98]. Significantly more tyrosol was secreted by *C. albicans* (range: 21.01 ± 0.76 to 53.40 ± 1.73 μM/1.6 × 10^7^–5.3 × 10^7^ cells/mL) and *C. tropicalis* (range: 41.21 ± 1.21 to 48.63 ± 3.83 μM/2.6 × 10^7^–2.7 × 10^7^ cells/mL) than by *Candida glabrata* (range: 1.3 ± 0.17 to 3.26±0.33 μM/2.7×10^7^–5.5×10^7^ cells/mL) or *C. parapsilosis* (range: 1.59 ± 0.29 to 3.04 ± 0.43 μM/1.7 × 10^7^–2.3 × 10^7^ cells/mL), suggesting a possible link with virulence [99]. Tyrosol plays a pivotal role in biofilm production, where it can stimulate hypha production of *C. albicans*, especially between two and six hours of biofilm development. *C. albicans* biofilms released at least 50% more tyrosol when compared to planktonic cells [96].

Regarding non-*albicans* species, tyrosol has been recognized as inducing the biofilm-forming ability of *C. auris* to grow as yeast or pseudohyphae [96]. Based on RNA-Seq analysis, tyrosol treatment resulted in 261 and 181 differentially expressed genes with at least a 1.5-fold increase or decrease in expression in *C. parapsilosis*, respectively; however, the initial adherence was not affected by the presence of tyrosol [43]. Interestingly, the ortholog of the *C. albicans CZF1* gene, which is a key transcription factor of biofilm development in *C. parapsilosis*, was upregulated following tyrosol exposure [43,100]. Nevertheless, Jakab et al. (2019) did not observe higher rates of biofilm formation in the presence of tyrosol [43]. In *C. parapsilosis*, tyrosol exposure overexpressed the active efflux pumps and caused an enhanced oxidative stress response, while inhibiting growth, ribosome biogenesis, and virulence. Surprisingly, its metabolism was modulated toward glycolysis and ethanol fermentation [43]. Monteiro et al. (2015) reported that tyrosol exposure did not induce increased adhesion in *C. glabrata* [101].

Regarding tyrosol related toxic effect, initial cytotoxicity was observed at concentrations of >10 mM, 3 mM, 5 mM and >15 mM for human gingival fibroblasts (GN61), human gingival epithelial cells (S-G), human salivary gland carcinoma cells (HSG_1_) and colon adenocarcinomas cell line (Caco-2), respectively [43,102].

### 5.2. Antimicrobial Activity of Tyrosol

Tyrosol is a relatively understudied molecule compared to farnesol in terms of potential antifungal or anti-biofilm activity; despite this, a few studies have examined the potential use of tyrosol in monotherapy or in combination with traditional antifungal agents against *Candida* species [36,72]. Arias et al. (2016) showed that tyrosol treatment at concentrations ranging from 100 to 200 mM exerted a significant reduction in metabolic activity against *C. albicans* and *C. glabrata* two-day-old oral biofilms, which was proportional to a reduction in cell number [103]. Do Vale et al. (2017) showed that tyrosol alone at concentrations of 50 and 90 mM demonstrated inhibition of the planktonic growth of *C. albicans* and *C. glabrata* cells, respectively [104]. However, tyrosol does not significantly reduce metabolic activity or the number of cells for one-day-old oral biofilms; in addition, the nature of interaction of tyrosol with chlorohexidine gluconate was indifferent. Nevertheless, 1.25 mM tyrosol with 0.00725 mM chlorhexidine gluconate showed a synergistic interaction in reducing the number of hyphae formed [104]. A combination of tyrosol and farnesol has been explored for oral *Candida* isolates for both planktonic and sessile growth. This combination showed synergy against *C. glabrata*, indicating that this combination may contribute to the development of oral care products against *Candida* species [105].

In another study, tyrosol showed anti-biofilm activity against denture-derived *C. albicans* isolates. However, it has been shown that the single use of tyrosol cannot decrease hydrolytic enzymes on oral *C. albicans* [106]. Shanmughapriya et al. (2014) observed that tyrosol treatment caused a 25% and a 50% reduction in intrauterine device-derived *Candida krusei* and *C. tropicalis* biofilm production at concentrations of 40 μM and 80 μM, respectively [107]. In addition, amphotericin B combined with tyrosol showed a remarkable inhibitory effect against these non-*albicans* biofilms. A concentration of 4 mg/L amphotericin B in the presence of 80 μM tyrosol exerted approximately 90% inhibition in biofilm formation [107]. Cordeiro et al. (2015) showed that the addition of tyrosol significantly reduced the MICs for amphotericin B, fluconazole, and itraconazole against planktonic *C. albicans* and *C. tropicalis* [108]. Furthermore, exogenous tyrosol alone was able to significantly reduce the biofilm formation of these species at concentrations ranging from 125 to 250 mM. At these concentrations, tyrosol decreased the metabolic activity of growing biofilms by approximately 24 and 30% for *C. albicans* and *C. tropicalis*, respectively. Reduction of metabolic activity was more pronounced when tyrosol was combined with traditional antifungal drugs including amphotericin B, fluconazole, and itraconazole. It should be noted that application of amphotericin B with tyrosol markedly decreased the metabolic activity of mature biofilms (35%) [108]. Kovács et al. (2017) reported that tyrosol may be used as an adjuvant agent with caspofungin or micafungin in alternative treatment strategies [109]. Regarding the in vivo antifungal effect of tyrosol, Jakab et al. (2019) reported that daily treatment with 15 mM tyrosol decreased the fungal tissue burden in their immunocompromised mouse model [43]. In this study, the expression of *ALS6*, which has a pivotal role in adhesion, was significantly reduced by tyrosol treatment. Furthermore, downregulation of the expression of *FAD2* and *FAD3* may also contribute to decreased virulence and kidney fungal burden. The well-documented antifungal effects exerted by tyrosol may be explained by the enhanced oxidative stress and the inhibition of virulence-related genes, growth, and ribosome biogenesis. In addition, tyrosol can alter the metabolism of *Candida* cells toward fermentation [43].

Data on the potential antibacterial effects of tyrosol remain scarce. Arias et al. (2016) found a potential anti-biofilm activity of tyrosol against *S. mutans* in single and mixed species biofilms with *C. albicans* or *C. glabrata* developed on acrylic resin and hydroxyapatite surfaces [103]. Their results may contribute to the development of innovative topical therapies focusing on biofilm-associated oral diseases. Abdel-Rhman et al. (2016) reported substantial antibacterial activity of tyrosol against *S. aureus*; moreover, tyrosol increased susceptibility to gentamicin, amikacin, and ciprofloxacin at subinhibitory concentrations ranging from 3.5 to 14.3 mM [110]. Tyrosol treatment can also influence *S. aureus* virulence, decreasing the production of protease and lipase enzymes and limiting the ability to form biofilms [110]. In the case of *P. aeruginosa*, tyrosol strongly inhibited haemolysin and protease production [111].

## 6. Future Remarks

Paradoxically, medical advancement has resulted in an increasing number of immunocompromised individuals susceptible to *Candida* infections. The incidence and mortality rate related to systemic *Candida* infections has remained unchanged, despite the advances in the field of antifungal therapy. Based on recent comprehensive epidemiological studies, the high incidence and mortality may be attributed to sessile *Candida* populations, namely biofilms, which show high resistance against environmental factors, immune responses, and traditional antifungal therapy. Although there is no definitive solution or highly effective therapeutic recommendation against *Candida* biofilms, there are many promising therapeutic strategies including antifungal “lock” therapy, photodynamic inactivation, and the use of natural products or synthetic peptides with antifungal activity. A further solution may be the utilization of quorum-sensing molecules alone or in combination with traditional antifungal agents; however, there are numerous open questions as to their exact action or the interaction between quorum-sensing molecules and the host. In addition, the full understanding of quorum sensing in non-*albicans* species has remained unelucidated. In this review, we provided an overview on the current status of studies focusing on anti-biofilm activity of farnesol and tyrosol. Hopefully, these in vitro and in vivo results can be implemented in therapeutic practice as soon as possible to overcome *Candida* biofilm-related infections.
